# Supplementary material for: Are carbon emissions trading and green financial instruments synergistic? -Comprehensive quantitative research based on content analysis
Source: PLoS One. 2024 Mar 7;19(3):e0298601. doi: 10.1371/journal.pone.0298601 (PMC10919601; doi:10.1371/journal.pone.0298601)
Supplement: S1 Data — (ZIP) [file pone.0298601.s001.zip › Data/地级市Z_F工作报告.zip!/║╙─╧/┬σ╤⌠╩╨/└┤╫╘╣1⁄2╓┌║┼í╛┬φ┐╦╩2╛▌═°í┐_2006.docx]

各位代表：

　　现在，我代表市人民政府，向大会作工作报告，请予审议，并请各位政协委员和列席人士提出意见。

　　一、2005年及“十五”工作回顾

　　2005年，在省委、省政府和市委的正确领导下，在市人大、市政协的监督、支持下，市政府和全市人民一道，全面贯彻科学发展观，积极构建和谐社会，锐意进取，扎实工作，圆满完成了市十二届人大二次会议确定的各项目标任务。

　　经济总量跃上新台阶。初步核算，全市生产总值达到1111.5亿元，较上年增长15.1%。其中，第一产业110.5亿元，增长7%；第二产业648.4亿元，增长17.3%；第三产业352.6亿元，增长13.6%。人均生产总值达到1.7万元。

　　工农业生产再创新高。规模以上工业增加值达到359.8亿元，实现销售收入1078亿元，实现利润52.6亿元，分别增长24.2%、39.1%和43.9%。粮食总产210.5万吨，是历史上第二个丰收年；肉、蛋、奶总产62.4万吨，增长21.9%。

　　投资、出口、消费全面增长。全社会固定资产投资完成480亿元，增长45.5%，其中城镇投资400.9亿元，增长48.4%。进出口总额9.6亿美元，增长27.9%，其中出口7.2亿美元，增长22.8%。实际利用外资1.14亿美元，增长28.4%；引进市外境内资金40.1亿元，增长12.6%。社会消费品零售总额340.7亿元，增长14.4%。

　　财政收支实现新突破。全部财政收入完成120.9亿元，增长32.3%，其中财政一般预算收入60.9亿元，增长41.7%。财政一般预算支出88.9亿元，增长41.5%。9县(市)和3个城市区财政收入超亿元，其中偃师、栾川、新安、伊川超5亿元，涧西、西工超2亿元，分别列全省县级和区级收入前8名。

　　就业和社会保障成效显著。城镇新增就业13.1万人；下岗失业人员再就业7.4万人，其中“4050”人员1.5万人。征缴各项社保基金18.5亿元、支出17.9亿元，16.1万名企业离退休人员养老金按时足额发放，10.3万人享受城市最低生活保障，7.3万名农村特困群众按月按标准享受救济。

　　城乡居民收入进一步提高。城市居民人均可支配收入达到10175元，增长12.7%；农民人均纯收入2903元，增长12.3%。城乡居民储蓄存款余额595.2亿元，比年初增加76.4亿元。

　　2005年各项目标任务的圆满完成，标志着我市经济社会发展实现了重要意义的跨越，使“十五”时期成为洛阳经济实力大幅提升、城乡面貌发生深刻变化、人民群众得到较多实惠的时期。

　　这五年，“千百亿”目标超额完成，综合经济实力显著增强。国民经济连续五年实现了较高的增长速度和较高的增长质量，各项主要指标均比“九五”末翻了一番还要多。生产总值突破千亿元大关，是2000年的2.6倍，年均增长13.5%，分别高出全国、全省4.1和2.1个百分点。全部财政收入突破百亿元，是2000年的2.6倍。五年完成全社会固定资产投资1354亿元，是“九五”的2.5倍。外贸出口和社会消费品零售总额分别是2000年的5.1倍和2.1倍。

　　这五年，农业结构实现重大调整，农村社会面貌发生显著变化。粮食总产连续3年保持在200万吨以上。烟叶种植面积跃居全省第一，烟叶生产成为农民致富的重要产业。畜牧养殖业迅猛发展，奶牛存栏跃居全省首位。农业产业化龙头企业发展到220家。劳务输出超过100万人，劳务经济成为农民增收的重要途径。相继实施了新安提黄、陆浑西干渠、龙脖灌区改造、孟西灌区等重点项目，五年新增有效灌溉面积18万亩，解决农村饮水困难和饮水安全32万多人。改造农村中小学D级危房2224所，2260个行政村实现“村村通”，改造建设乡镇卫生院117所，农村基础设施得到明显改善。五年搬迁深山区贫困人口3744户、1.5万人，11.6万人告别贫困。

　　这五年，四大产业基地迅速壮大，工业支撑能力显著提升。凝聚全市力量，坚定不移地实施工业强市战略，努力建设先进制造业、铝工业、电力工业和石化工业基地。五年实施千万元以上工业项目903个，完成投资475.3亿元。先进制造业快速壮大，一拖、洛玻、中信重机等传统企业竣工项目达到140个。铝工业从无到有，在建氧化铝生产能力120万吨，达产电解铝58万吨，达产和在建铝精深加工生产能力达60万吨，煤电铝及铝加工产业链趋于完善。电力工业五年新增发电装机容量260万千瓦，在建项目完成后可使装机总容量达到632万千瓦。石化工业已形成从炼油到PTA、聚酯、长丝、短纤的产业链，附加值明显提高。全市销售收入超50亿元企业达到4家，洛阳石化达到212亿元。规模以上工业增加值、销售收入和利润分别是2000年的3.1倍、3.3倍和16.1倍；工业经济综合效益指数达到172.6，比2000年提高96.3个百分点；工业增加值占生产总值的比重达到50.9%，对经济增长的贡献率达到59.9%。

　　这五年，基础设施建设快速推进，城镇化进程显著加快。以洛河为轴线、南北对应发展，跨过洛河建新城，实现了洛阳城市建设的重大跨越。五年投入城建资金396亿元，是“九五”的近40倍。新建改扩建城市道路334条254公里，建成了洛浦公园、王城大道等一批重大项目，形成了龙门西山、周山、小浪底、上清宫四大森林公园，开工建设北邙绿色屏障和隋唐城遗址植物园，治理涧河、?『印⒅兄萸?、大明渠和秦岭防洪渠，实施地块开发项目125个。五年完成交通投资104亿元，环城高速全线贯通，洛栾快速通道、南部山区大环线建成通车，新建改建县乡公路1100公里。9县(市)县城面貌焕然一新，一批污水处理和垃圾处理项目正在实施。投入26.4亿元改造小城镇138个，有力地带动了农村经济社会发展。全市城镇化率达到38%，比2000年提高8个百分点。

　　这五年，旅游强市战略有效实施，服务业发展水平显著提高。以旅游业为龙头，大力发展服务业，形成了牡丹花会、河洛文化旅游节、伏牛山滑雪节等节会品牌，成功入选中国十大最佳魅力城市和欧洲人最喜爱的中国十大旅游城市，白云山被评为十个“中国最美的地方”之一，接待海内外游客突破2100万人次，旅游总收入突破100亿元。商贸流通业升级步伐加快，丹尼斯、王府井、家乐福等相继入驻，一批超亿元项目进展顺利。房地产业发展势头强劲，五年完成房地产开发投资141亿元，竣工1138万平方米。金融保险、邮电通信、社区服务等服务业快速发展。

　　这五年，非公有制经济加快发展，县域经济实力迅速壮大。积极完善政策，优化环境，大力实施骨干带动工程，非公有制企业税收达到45亿元，是2000年的5倍。营业收入超亿元企业达到66家。以县域工业为支撑，以非公有制经济为主体，因地制宜发挥比较优势，大力发展特色产业，县域经济发展实现重大突破，一些贫困县一改落后面貌，经济总量和财政收入跃居全省前列。9县(市)生产总值占全市比重由2000年的52.9%提高到61.3%，规模以上工业增加值占全市比重由26.5%提高到48.5%，财政一般预算收入占全市比重由38.3%提高到46.8%。

　　这五年，改革开放取得重大进展，经济发展活力显著增强。国有大中型企业产权制度改革取得突破，洛铜引进中铝公司进行战略投资，洛耐并入中钢集团序列，轴研科技成功上市。10户重点企业的125个辅业单位完成分离。一批企业学校、医院实现移交。203户市属中小企业改革基本完成。全面取消农业税，长达2600多年的古老税种退出历史舞台。农村信用社改革、乡镇机构改革、矿产资源整合和财政、交通、市政公用等改革全面推进。大力实施开放带动战略，不断创新招商方式，全市外商投资企业达到709家，五年实际利用外资3.6亿美元，利用市外境内资金145.9亿元。外贸出口19.2亿美元，130多个“洛阳制造”产品进入国际市场。

　　这五年，社会事业全面进步，人民生活水平显著改善。五年新增城镇就业42.5万人，下岗失业人员再就业22.1万人。社会保障覆盖面不断扩大，“两个确保”全面落实，养老保险市级统筹有序推进，新型农村合作医疗试点运行平稳。城市居民人均可支配收入和农民人均纯收入分别是2000年的1.8倍和1.5倍。居民消费结构明显升级，城市居民人均居住面积达到22.2平方米，通信、汽车、电脑等成为新的消费热点。积极推进科技创新，五年共有520个项目列入国家、省科技计划，10个项目列入国家“863计划”。获得国家级科技进步奖6项、省级95项。落实免杂费、免书本费和补助寄宿生生活费政策，惠及农村贫困家庭学生64.4万人次。普通高招连创历史新高，新区大学园区初具规模。市、县疾病控制中心、卫生监督中心投入使用。打造了《河洛风》、《牡丹魂》等文化精品，青年宫文化中心、新区体育中心、歌剧院等一批标志性文化项目顺利实施。加强超标排污企业治理，城市空气质量良好天数达到265天。民族宗教、计划生育、体育、物价、统计、审计、文物、外事、侨务、人防、气象、地方志等都取得了新的成绩。

　　精神文明和民主法制建设取得新的成效。扎实开展保持共产党员先进性教育活动，广泛开展“道德规范进万家，诚实守信万人行”活动。积极开展文明景区、文明城市创建活动，荣获“全国创建文明城市工作先进市”。第四次荣获全国双拥模范城称号。顺利完成村民委员会换届选举。坚持依法行政，五年提请市人大审议通过地方性法规23件，颁布规章34件。自觉接受人大及其常委会的法律监督、工作监督和政协民主监督，认真办理人大代表议案、建议和政协委员提案。加强社会治安防控体系建设，依法做好信访稳定工作，荣获“全国综治先进市”称号。

　　各位代表：过去的五年，是我市发展史上不平凡的五年，是全市人民开拓创新、奋发有为的五年。五年来，全市上下积极探索加快洛阳发展的有效途径，坚持在宏观调控中抢抓发展机遇，坚持在结构调整中培育发展后劲，坚持在完善基础设施中加快城乡建设，坚持在改革开放中增强发展活力，坚持在统筹协调中促进社会全面进步，有力地推动了全市经济社会的持续快速健康发展。这五年积累的宝贵经验和探索的正确发展思路，需要我们在今后工作中继续发扬和长期坚持。

　　各位代表：这些成绩的取得，是省委、省政府和市委正确领导的结果，是人大、政协积极支持的结果，是全市人民抢抓机遇、共同奋斗的结果。我代表市人民政府，向全市广大工人、农民、知识分子、干部，向人大代表和政协委员，向驻洛部队指战员、武警官兵和公安干警，向各民主党派、人民团体和离退休老同志，向所有关心支持洛阳发展的各界人士，致以崇高的敬意和衷心的感谢！

　　在肯定成绩的同时，我们还清醒地看到，经济社会发展中仍然存在一些深层次矛盾和问题：经济高速增长较多地依赖生产要素的高投入和资源的高消耗，经济发展与人口、资源、环境的矛盾亟待解决；城乡发展不平衡问题仍较突出，农民增收的长效机制尚未形成，城市低收入群体生活困难；企业产权制度改革尚待进一步深化，经济效益有待进一步改善；经济发展软环境仍需优化，利用外资规模和对外开放水平需要进一步提高；安全生产存在薄弱环节，影响社会稳定的问题依然突出。这些问题要在今后工作中认真解决。

　　二、“十一五”发展的基本思路和奋斗目标

　　“十一五”时期是我市全面建设小康社会的关键时期，我市经济社会发展的基本思路是：以邓小平理论和“三个代表”重要思想为指导，以科学发展观统领经济社会发展全局，坚持把发展作为第一要务、把改善人民生活作为出发点和落脚点，大力实施“工业强市、旅游强市”战略，深化改革，扩大开放，进一步加快工业化、城镇化，积极推进社会主义新农村建设，加快结构调整，转变增长方式，强化自主创新，完善体制机制，促进社会和谐，实现经济社会又快又好发展，把洛阳打造成国家新型工业基地和中西部地区最佳人居环境城市，努力再造一个新洛阳。

　　“十一五”经济社会发展的主要奋斗目标是：

　　――实现主要经济指标再翻一番。到2010年生产总值达到2200亿元以上，其中工业增加值1200亿元以上；全部财政收入240亿元以上，其中一般预算收入120亿元以上，均比2005年翻一番以上。

　　――加快建设国家新型工业基地。着力提高自主创新能力，全力打造先进制造业、铝工业、能源电力、石化工业、硅产业、钼钨钛产业等六大产业基地，到2010年规模以上工业销售收入达到2200亿元以上，利税达到180亿元以上。

　　――加快建设社会主义新农村。坚持工业反哺农业、城市支持农村，加快建设生产发展、生活宽裕、乡风文明、村容整洁、管理民主的新农村。“十一五”期间，力争改扩建农村公路7700公里，农村新型合作医疗覆盖率95%，沼气普及率30%，农民人均纯收入年均增长6.5%，基本完成现有贫困人口脱贫。

　　――加快建设中西部地区最佳人居环境城市。进一步加快城市基础设施建设，完善城市功能，实施“碧水蓝天青山绿地”工程，建成国家卫生城市，争创国家环保模范城市，建设国际性旅游城市。到2010年，城镇化率达到48%，城市污水集中处理率达到90%，垃圾无害化处理率97%，空气质量良好天数292天以上，接待国内外游客3000万人次以上。

　　――加快建设开放型城市。坚持以开放促改革、促发展，全方位扩大对外开放，五年累计利用外资10亿美元以上，引进市外境内资金400亿元以上。“十一五”末外贸出口达到14亿美元。非公有制经济比重达到58%。

　　――加快构建和谐洛阳。坚持以人为本，更加关注民生，让人民群众充分享受改革发展成果。五年新增城镇就业40万人以上，养老、失业、医疗保险覆盖面达到95%以上。坚持节约发展、清洁发展、安全发展和可持续发展，积极建设资源节约型、环境友好型社会，单位生产总值能耗比“十五”末降低20%左右。加快发展社会事业，健全公共服务体系，促进人的全面发展和社会全面进步。

　　《洛阳市国民经济和社会发展第十一个五年规划纲要(草案)》及其说明已印发各位代表，请予审议。

　　三、2006年工作的主要目标和任务

　　今年是“十一五”规划开局之年，全市经济社会发展的主要预期目标是：生产总值增长13.5%；全社会固定资产投资增长18%以上；社会消费品零售总额增长15%；外贸出口增长18%；财政一般预算收入增长16%；城市居民人均可支配收入增长10%；农民人均纯收入增长8%；单位生产总值能耗降低4%；人口自然增长率控制在6.5‰以内；城镇登记失业率控制在4.5%以内。

　　围绕上述目标，重点抓好以下工作：

　　(一)以建设新农村为主线，促进农村经济社会全面发展

　　积极推进社会主义新农村建设。围绕促进产业繁荣、增加农民收入、改善基础设施、保护生态环境、整洁村容村貌、打造文明乡风、完善社会保障、创新发展机制、提高农民素质、搞好民主管理、建设五好班子、实现社会和谐等重点工作，认真编制新农村建设规划，全面开展“千村整治百村示范”工程，优先支持城市远景规划区、县城及产业聚集区内的新农村建设。发挥示范带动作用，鼓励基层创造性工作，积极探索社会主义新农村建设的有效途径。

　　重点推进农村基础设施建设。投资4.7亿元，完成715个村1798公里“村村通”工程。投资2.5亿元，建成孟西灌区一期，搞好陆浑、玉马等水库除险加固和禹门河、金牛岭等电源工程。积极服务西霞院等大型水利工程建设。搞好灌区修复配套，新增有效灌溉面积3万亩、节水灌溉4万亩。治理水土流失200平方公里，改造中低产田3.3万亩。筹措4750万元，解决13.2万人安全饮水，鼓励有条件的地方发展农村自来水。筹措1300万元，支持发展户用沼气6万座。大力实施退耕还林、天然林保护工程和北邙绿色生态屏障工程，完成荒山造林20万亩、高标准通道绿化400公里、绿化示范村400个。积极推进敬老院、文化大院等公益设施建设，使农民享受更多的公共服务。加快农村商业设施建设，鼓励城市连锁超市向农村延伸。

　　扎实推进农业结构调整。优化粮食种植结构，提高粮食单产，力争优质小麦达到185万亩，优质玉米稳定在118万亩，粮食总产稳定在200万吨以上。筹措8000万元，完成22个种烟大乡的烟水配套工程，力争烟叶面积突破30万亩，烟叶特产税达到7500万元以上。

　　千方百计增加农民收入。坚持“多予、少取、放活”方针，认真落实各项支农惠农政策，对种粮农民的直接补贴、良种补贴和大型农机具购置补贴争取达到6000万元以上。继续实施农村百万劳动力转移工程，着力提高务工农民择业能力，实现劳务输出120万人，劳务创收40亿元。不断提高农民的组织化程度，争取农业产业化龙头企业达到230家、各类专业合作经济组织240家。加强农民实用技术培训，完成农民培训63万人。筹措6400万元，完成搬迁扶贫3000户、1.2万人，力争3年内完成9603户3.8万人的搬迁扶贫任务。妥善解决小浪底库区新增移民和陆浑水库回流移民生活问题。加强涉农收费和价格监管，防止农民负担反弹。

　　(二)以提高自主创新能力为核心，加快产业基地建设和产业升级步伐

　　加大项目实施力度，加快重点项目建设，促进固定资产投资快速增长，确保全社会固定资产投资达到566亿元以上。

　　着力建设六大产业基地。全年计划实施千万元以上工业项目184个，其中新开工91个，竣工120个；项目总投资483亿元，当年完成投资160亿元。不断提升先进制造业水平。确保一拖3万台大中马力轮式拖拉机、中信重机大型干法水泥和矿山设备等37个项目竣工投产；开工洛铜10万吨高精度电子铜板带和洛玻高档低辐射镀膜玻璃等26个项目；积极推进一拖2万台重型柴油机等14个项目的前期工作。大力发展铝精深加工。确保新安电力120万吨氧化铝一期，中铝河南铝业20万吨热轧、20万吨冷轧等9个项目竣工投产；新开工恒基铝业20万吨4A沸石等6个项目；积极推进伊川电力铁路高速机车车厢板材等项目的前期工作。积极发展能源工业。确保华润2×60万千瓦机组、正村120万吨煤矿等7个项目竣工投产；积极推进台塑2×60万千瓦机组、新区2×30万千瓦机组等项目的前期工作。抓好“西气东输”、“川气入豫进洛”的争取工作，积极引进管道天然气。加快壮大石化工业。确保黎明化工院推进剂、吉利5万吨轻烃燃气一期等17个项目竣工投产；开工洛阳石化800万吨炼油扩能等18个项目；继续做好45万吨PX、60万吨PTA项目的前期工作。加快培育硅产业。积极推进偃师1000吨多晶硅项目建设，确保尚德30兆瓦太阳能电池、阿特斯100兆瓦太阳能电池一期等3个项目竣工投产；抓紧开工洛龙科技园2000吨多晶硅项目；进一步加快硅产业链项目的规划和建设。加快开发钼钨钛产业。确保新安5000吨海绵钛等14个项目竣工投产；新开工汝阳金堆城1.5万吨钼选厂、洛钼集团4万吨氧化钼及钼冶炼等11个项目；积极推进725所万吨钛合金深加工、嵩县万吨钼选厂等项目的前期工作。

　　着力增强自主创新能力。按照自主创新、重点跨越、支撑发展、引领未来的方针，大力提高原始创新、集成创新和引进消化吸收再创新能力，用技术创新支撑新型工业基地建设。引进国家级重点实验室2个，新建市级研发中心30个，申报高新技术企业50家。围绕省部共建洛阳装备制造业基地，开展重大装备、新型材料、信息技术等领域的技术攻关，重点加强多晶硅、太阳能光伏电池等产业的攻关，形成一批具有自主知识产权的关键技术和产品。大力实施名牌战略，建立企业争创名牌的奖励机制，努力使更多产品进入国家、省名牌行列。发挥科研院所优势，支持科技成果就地转化和产业化，实现技工贸总收入90亿元。坚持人才强市战略，完善和落实吸引人才的政策措施，造就创新人才队伍。高度重视和关爱企业家，培养壮大优秀企业家队伍。

　　着力促进工业集聚发展。围绕洛阳优势产业，进一步发展特色产业和关联产业，拉长产业链条，培育产业集群，提高产业集聚度。加快工业园区建设，完善基础设施，增强综合配套能力，积极承接产业转移，使其成为工业发展的重要支撑。充分发挥洛阳高新区、洛阳经济开发区、洛阳工业园区和洛龙科技园、吉利科技园等园区的平台作用，抓紧项目的引进和实施，努力壮大产业规模。积极推广使用标准化厂房，构建中小企业发展新平台。

　　着力加强经济运行协调。搞好煤电油气运供需衔接，确保重点企业、重点项目需要。加强扭亏增盈工作，重点抓好盈利大户增盈和亏损大户的扭亏。推广中信重机经验，深化企业三项制度改革，最大限度地降低成本，减少两项资金占用。管好用好财政贴息资金，积极争取国家、省资金和银行贷款，加大对重点企业的服务力度。力争销售收入超50亿元企业达到7家，其中超100亿元企业4家、超260亿元企业1家。

　　(三)以旅游业为龙头，进一步发展壮大服务业

　　全面提升旅游业。坚持旅游强市战略，积极推进旅游与文化、商贸业的结合，提高旅游综合效益。以所有权、管理权和经营权分离为突破口，推动旅游景区企业化经营、市场化运作。整合旅游资源，培育精品线路，逐步形成历史文化、生态山水、旅游节会、休闲度假等旅游品牌。进一步完善旅游交通体系，努力增加航空班次、旅游专列。切实抓好白马寺印度佛殿等项目建设。精心办好牡丹花会、河洛文化旅游节、伏牛山滑雪节等节庆活动。加大市场开发力度，积极策划国外旅游促销，努力实现入境游的快速增长。实行贷款贴息等鼓励政策，做大做强旅游骨干企业。力争全年接待游客2400万人次，其中境外游客18万人次。

　　加快发展文化产业。按照为群众服务、为旅游业服务的思路，繁荣文化产业，丰富群众文化生活。深化文化体制改革，尽快使经营性文化事业单位改制为企业。加快洛阳歌剧院、体育中心二期等文化体育设施建设。繁荣发展新闻出版、广播影视和文化艺术，培育健康有序的文化市场。办好新组建的洛阳豫剧院。积极引进大型赛事，搞好新区体育场馆的市场化运作。

　　大力发展商贸流通业。加快国贸中心、新都汇、中原康城等重大项目建设，完善商业网点，力争新增营业面积34万平方米。大力发展物流配送、电子商务等现代流通方式，促进传统商业企业改造升级。着力培育大型流通企业，增强对周边地区的辐射能力。加快发展专业特色街和社区商业，进一步繁荣市场，引导和拉动消费。

　　进一步激活房地产业。支持我市房地产企业通过兼并、收购、重组壮大规模，培育房地产骨干企业。鼓励外地房地产企业来洛开发，吸引外地居民来洛购房。力争房地产开发施工面积达到600万平方米。深化住房制度改革，活跃住房二级市场。全面加强和改善物业管理，营造美好生活环境，为住房消费提供良好条件。

　　积极发展金融等服务业。发挥金融对投资和消费的支持作用，积极开发和利用新型金融服务产品。严厉打击逃废金融债务行为，构建良好金融生态环境。支持企业通过股票、债券、信托等途径直接融资。积极培育保险市场。全面发展信息、法律、会计等中介服务业。加快发展社区服务、交通运输、邮电通信等服务业。

　　(四)以基础设施建设为依托，进一步加快城镇化进程

　　加快推进新区建设。全年计划完成投资75亿元，基本完成新区道路、配套管网、园林绿化等工程。确保牡丹大道、关林路等12条道路如期竣工；新开工开元大道东西延长线等18条道路。加快隋唐城遗址植物园建设，完成新区水系工程，形成水面1900亩。全面完成村庄拆迁，村民安置小区全部建成入住。积极推进河科大、工业高专等项目建设，开工建设新区医院和普通高中。加快土地出售步伐，推进重点地块开发。完善商业超市、文化休闲、餐饮娱乐等设施，健全综合服务功能，实现成规模、聚人气、出形象目标。

　　积极推进洛北城区改造。全年计划完成投资63亿元，突出抓好“一河两渠”治理、定鼎北路、唐宫东路、社区道路改造、周王城广场二期、洛浦公园美化亮化、供水供电管网改造、重点区域亮化等45个市政公用项目。实施地块改造项目138个，确保世纪金苑、凯旋花园等41个项目年内竣工。完善城区改造规划，推进城中村改造。优先发展城市公交，加快建设和完善公交场站。

　　大力推进县城和小城镇建设。高起点搞好县城规划，加快道路、供水、供电、通信等基础设施建设，努力建设设施完备、特色鲜明、环境优美、管理有序的现代化县城。进一步完善小城镇功能，提高经营管理水平，构筑二、三产业发展平台，招商引资培育产业支撑，不断增强辐射带动能力。

　　继续加快交通建设。确保完成投资20亿元以上，重点抓好连霍高速改造和洛阳至南阳高速建设。完成栾川方村至卢氏界等5个干线公路项目，新开工洛界高速汝阳连接线等5个项目。新建改建县乡公路112公里、乡村客运站171个，行政村通客车率达到88%。积极支持郑州-西安高速铁路客运专线和焦枝铁路电气化建设。

　　(五)以非公有制经济为支撑，促进县域经济再上新台阶

　　加快发展非公有制经济。认真落实《国务院关于鼓励和引导个体私营等非公有制经济发展的若干意见》，营造非公有制企业发展的良好环境。加强融资担保、创业辅导等社会化服务体系建设，引导金融机构加大对中小企业的信贷支持。支持非公有制企业参与国有企业改革，拓宽非公有制经济发展空间。依托六大产业基地，大力发展配套企业，培育产业集群，做大做强优势企业。力争非公有制企业营业收入达到1270亿元，超亿元企业达到80家以上。

　　进一步壮大县域经济。总结经验，完善政策，引导县(市、区)发展特色经济，推动县域工业相对集中规划和布局，因地制宜发展工业园区，培育特色产品和产业集群。完善城镇公共服务设施，加快现有企业改制，加大招商引资力度，促进资金、技术、人才等生产要素快速集聚。着力抓好项目建设，做大县域骨干企业，大力培育新的经济增长点，推动各县(市、区)经济实力实现新的提升。

　　(六)以构建和谐洛阳为目标，大力发展各项社会事业

　　认真落实科教兴洛战略。强化政府主导，加大科技投入，建设创新型城市，争取进入全国科技进步先进市行列。巩固提高“两基”成果，农村义务教育“两免一补”争取达到9400万元。投入950万元，提高农村中小学生均公用经费标准。安排专项资金，为贫困地区中小学统一更换课桌凳。建立中小学危房改造长效机制。大力发展高中阶段教育，加快发展职业教育和高等教育，支持洛阳大学、洛阳工业高专联合升本，支持组建洛阳医学高等专科学校。

　　加快完善公共卫生体系，积极解决看病难、看病贵问题。加大公共卫生投入，加强重大疫病预防控制。投入4080万元，完成76所乡镇卫生院国债项目建设。进一步搞好乡镇卫生院和农村卫生所建设，积极发展城市社区卫生服务，全面实施城乡医疗救助制度。搞好新安、偃师、嵩县、宜阳新型农村合作医疗试点，争取早日在全市全面施行。整顿医疗秩序，规范药品价格，进一步加强医院管理，全面提高医疗服务水平。

　　全面发展文化、体育等各项社会事业。充分发掘河洛文化资源，大力发展文化事业。加快广播电视“村村通”工程，扩大农村广播电视和信息网络覆盖面。全面启动有线电视数字化整体转换工程。积极推进县级图书馆、文化馆建设。加强五大遗址保护，做好文物保护利用工作。积极备战省十运会，深入开展全民健身活动。严格落实计划生育政策，稳定低生育水平。加强统计基层基础建设，认真组织第二次全国农业普查。全面做好外事、侨务、人防、气象、地方志等工作。

　　进一步加强就业和社会保障。全面落实小额贷款、社保补贴、税费减免等促进就业再就业的政策措施，力争城镇新增就业9万人，失业人员再就业3.5万人，其中“4050”人员1.3万人。依法扩大社会保障覆盖面，力争养老保险扩面2.8万人，征缴9.3亿元，清欠7000万元，确保企业离退休人员养老金按时足额发放。健全城乡低保，扩大农村“五保户”集中供养，筹措1.2亿元，救助低收入困难群众21万人。认真解决进城务工人员和失地农民的社会保障问题。继续抓好拖欠工程款和农民工工资清欠工作。

　　切实加强环境保护，努力实现清洁发展。认真落实创建目标责任制，力争上半年通过国家卫生城市考核，积极争创国家环保模范城市。提高5000万元环保专项资金使用效益，重点用于大气和水污染治理，确保全年城市空气质量良好天数达到270天以上，伊洛河出境水质达到省定标准。加强超标排污企业治理，坚决淘汰、搬迁污染企业，加快火电企业脱硫设施建设。按期完成涧河、瀍东、新区、吉利区和9县(市)污水处理厂建设。

　　大力发展循环经济，努力实现节约发展。认真做好新一轮土地利用总体规划修编工作，提高土地集约利用水平。完善政策措施，积极发展循环经济。从严控制高耗能、高耗材、高耗水产业，淘汰落后生产能力。整顿矿产资源开发秩序，推进煤、铝矾土、钼、黄金等资源整合。切实保护基本农田，逐步禁止生产实心黏土砖，积极推广新型墙体材料。

　　高度重视并切实抓好安全生产和社会稳定。认真落实安全生产责任制，全面开展专项整治，加强重大危险源监管和安全生产隐患的排查、整改，遏制重特大事故发生。整顿和规范市场经济秩序，加强食品药品监管，严厉打击制售假冒伪劣产品、价格欺诈等违法犯罪活动。认真做好人民群众来信来访工作，积极预防、妥善处置群体性事件和突发性事件。巩固“全国综治先进市”成果，强化社会治安综合治理，完善社会治安防控体系，努力建设平安洛阳。

　　(七)以产权制度改革为重点，推动各项改革向纵深拓展

　　继续深化国有企业改革。加快国有大中型企业产权制度改革，推进股份制改造，着力解决出资人监管不到位和法人治理结构不完善等问题。鼓励国有大型企业通过增资扩股、股权转让等方式引进战略投资者，争取国有工业企业基本实现产权多元化。基本完成国有大中型企业主辅分离和企业学校移交，加快企业医院分离工作。巩固中小企业改革成果，重点抓好资产变现、职工安置和恢复生产工作。支持有条件的企业规范上市，推进上市公司股权分置改革。加强国有资产监督管理，确保国有资产保值增值。

　　积极深化财税体制改革。深化部门预算、国库集中支付、政府采购等改革，加强非税收入管理，提高收入质量和资金使用效益。完善乡财县管，保障村级组织正常运转，逐步建立地方公共财政体系。强化税收征管，加大稽查力度，严厉打击偷逃税行为。加强税源建设，认真开展税收分析评估，在依法征收、应收尽收的同时使企业充分享受税收优惠政策。

　　全面加快重点领域改革。推进投融资体制改革，落实企业投资自主权。推行工资制度改革，落实公务员津贴补贴政策。巩固乡镇机构改革成果，促进乡镇职能转变。加快公共产品价格改革，确保物价稳定。继续做好农村信用社清收不良贷款工作。基本完成经营性事业单位企业化改制。进一步深化城建、交通、水利等方面改革。

　　(八)以招商引资为主战略，全面提升对外开放水平

　　努力实现招商引资新突破。坚持把招商引资放在经济工作首位，进一步加强领导，完善机制，强力推进，务求实效。结合我市产业现状和“十一五”规划的重大项目，坚持领导带队招商、驻地招商、会展招商、以商招商等行之有效的招商方式，有针对性地开展招商引资。强化企业的招商引资主体地位，支持企业拿出优质资产、优势项目吸引合作伙伴。认真组织参加重要经贸洽谈活动。完善招商引资考核办法，加强资金到位率和项目开工率的考核。力争实际利用外资1.5亿美元以上，利用市外境内资金60亿元以上。

　　努力保持外贸出口快速增长。进一步加强对企业的指导和服务，培育骨干出口企业，大幅度增加自主出口企业。优化出口商品结构，巩固传统大宗产品出口，促进机电产品和高新技术产品出口，力争全年外贸出口8.5亿美元以上。积极参与国际合作与竞争，扩大对外工程承包，带动劳务和设备输出。

　　(九)切实加强精神文明和民主法制建设

　　认真落实《公民道德建设实施纲要》，深入开展公民基本道德规范教育，树立以“八荣八耻”为具体内容的社会主义荣辱观，弘扬诚实守信、尊老敬贤、见义勇为的良好风尚。以伦理、心理、生理教育为载体，加强未成年人思想道德建设。扎实开展文明城市、文明景区和农村文明村镇、文明家庭创建活动，继续开展法律、科技、文体、卫生进社区和文化、科技、卫生“三下乡”活动。深入开展国防教育，关心支持驻洛部队、武警和民兵预备役建设。大力开展双拥共建活动，争创全国“双拥模范城”五连冠。

　　坚持依法治市，深入开展普法教育，提高全民法律素质。自觉接受人大及其常委会的法律监督、工作监督和政协民主监督，严格执行人大及其常委会的决议、决定，支持人大代表依法行使职权，认真办理人大代表议案、建议和政协委员提案。广泛听取各民主党派、工商联和无党派人士的意见和建议。充分发挥工会、共青团、妇联等人民团体的桥梁纽带作用。全面贯彻党的民族宗教政策，做好民族宗教工作。加强法律援助，依法保护公民特别是妇女儿童、残疾人和未成年人合法权益。

　　(十)全面加强政府自身建设

　　努力建设服务型政府。牢记执政为民宗旨，认真解决广大群众最关心、最直接、最现实的利益问题。政府各部门都要就服务事项、服务标准、服务期限、服务质量向社会公开承诺。完善政府新闻发布和信息公布制度，建好政府门户网站，办好“市长热线”，更好地为基层、为企业、为市民服务。

　　努力建设法治政府。全面落实行政许可法、公务员法和国务院全面推进依法行政实施纲要，严格按照法定权限和程序办事。强力推行行政执法责任制和执法过错追究制，切实做到执法有保障，有权必有责，用权受监督，侵权须赔偿，违法要追究。

　　努力建设廉洁政府。深入开展廉政建设和反腐败斗争，重点治理工程建设、土地出让、产权交易、医药购销和政府采购等领域的商业贿赂问题。坚决纠正一切行业不正之风，当前要着力解决教育乱收费、医疗高收费等突出问题，以廉政建设的实际成效取信于民。

　　努力建设效能政府。加强机关效能建设，全面推行绩效考核，促进政府工作提速。落实首问责任制、限时办结制和失职追究制，做到有报必理、有理必果、快办办好，健全有序高效的工作机制。

　　努力建设信用政府。坚持求真务实，加强政务督查，全面兑现政府对人民群众的承诺，落实对企业和外来投资者的优惠政策，言行一致，履约践诺，提高政府公信度。

　　努力建设学习型机关。加强公务员的教育和管理，大兴调查研究之风，形成自觉学习、善于学习的良好风气，使全体公务员熟知法律、政策和市情，加强政策和机遇研究，提高工作水平。

　　各位代表，站在新的历史起点上，展望洛阳的未来，我们满怀信心和希望。让我们在以胡锦涛同志为总书记的党中央领导下，高举邓小平理论和“三个代表”重要思想伟大旗帜，全面贯彻落实科学发展观，振奋精神，团结拼搏，为实现再造一个新洛阳的宏伟目标而努力奋斗！
